# Neoadjuvant chemoradiotherapy vs. direct surgery for locally advanced esophageal cancer: a meta-analysis

**DOI:** 10.3389/fsurg.2026.1788125

**Published:** 2026-05-20

**Authors:** Lun Li, Lei Liu, Cong Yu, Shuzhu Chen

**Affiliations:** 1 Department of Thoracic Surgery, Qingdao Hospital, University of Health and Rehabilitation Sciences (Qingdao Municipal Hospital), Qingdao, China; 2 Department of Thoracic Surgery, The Affiliated Hospital of Qingdao University, Qingdao, China; 3 Department of Gastroenterology, Qingdao Hospital, University of Health and Rehabilitation Sciences (Qingdao Municipal Hospital), Qingdao, China

**Keywords:** esophageal cancer, neoadjuvant chemoradiotherapy, meta-analysis, overall survival, R0 resection, randomized controlled trial

## Abstract

**Objective:**

This meta-analysis systematically compares neoadjuvant chemoradiotherapy (nCRT) plus surgery vs. surgery alone for locally advanced esophageal cancer.

**Methods:**

Randomized controlled trials (RCTs) were searched in PubMed, Embase, and Cochrane Library from inception to March 2025. Study selection, data extraction, and risk of bias assessment (using ROB 2.0) were performed independently. Pooled hazard ratios (HRs) for overall survival (OS) and disease-free survival (DFS), and odds ratios (ORs) or relative risks (RRs) for R0 resection, pathological complete response (pCR), postoperative complications, and mortality were calculated using Stata 18.0. Heterogeneity was assessed with the I^2^ statistic.

**Results:**

Seven RCTs involving 1,168 patients were included. nCRT plus surgery significantly improved OS (HR = 0.68, 95% CI 0.59–0.77, *P* < 0.001) and DFS (HR = 0.59, 95% CI 0.50–0.67, *P* < 0.001) compared to surgery alone. The R0 resection rate showed a non-significant increase (OR=1.40, 95% CI 0.72–2.08, *P* = 0.32), while the pCR rate was significantly higher with nCRT (OR=8.42, 95% CI 4.88–14.52, *P* < 0.001). Postoperative complications were significantly higher in the nCRT plus surgery group (RR = 1.18, 95% CI 1.05–1.33, *P* = 0.006), while treatment-related mortality showed no significant difference (RR = 1.32, 95% CI 0.98–1.78, *P* = 0.065). Heterogeneity was low for OS and DFS (I^2^ = 0%) and moderate for R0 resection (I^2^ = 58.42%). Potential publication bias was detected for R0 resection.

**Conclusion:**

nCRT followed by surgery significantly enhances survival outcomes and pCR rate, but does not significantly improve the R0 resection rate compared with surgery alone, without increasing mortality risk.

**Systematic Review Registration:**

https://www.crd.york.ac.uk/prospero/display_record.php?ID=CRD42025125423, PROSPERO CRD420251251423.

## Introduction

Esophageal cancer is a common malignant tumor of the digestive tract worldwide, characterized by high incidence and mortality rates and a poor prognosis (1, 2). The treatment of locally advanced esophageal cancer (LAEC) remains a significant clinical challenge (3). For a long time, radical esophagectomy alone was the standard treatment for LAEC; however, its long-term survival rate is suboptimal, with high risks of local recurrence and distant metastasis (4). To improve the prognosis of LAEC patients, the multimodal approach of neoadjuvant chemoradiotherapy (nCRT) followed by surgery has become a research focus (5). This strategy aims to reduce tumor size, downstage the disease, increase the likelihood of radical resection, and eradicate micrometastases preoperatively (6). Several randomized controlled trials (RCTs), such as the CROSS and NEOCRTEC5010 studies, have demonstrated significant survival benefits with nCRT (7). Nevertheless, heterogeneity persists among different studies regarding survival outcomes and surgical radicality, such as R0 resection rates (7). Although previous meta-analyses have synthesized this evidence, the recent updates of long-term follow-up data from high-quality RCTs necessitate further integration of the evidence. Furthermore, prior meta-analyses often did not perform subgroup analyses stratified by tumor histology (squamous cell carcinoma vs. adenocarcinoma) or geographic region, nor did they systematically explore how different neoadjuvant chemotherapy regimens or radiation doses might affect heterogeneity in R0 resection rates. The present study addresses these gaps by conducting prespecified subgroup analyses and sensitivity analyses (8, 9). Therefore, this study aims to conduct an updated meta-analysis, via systematic retrieval and rigorous assessment, to compare the efficacy (overall survival, disease-free survival, R0 resection rate) of nCRT plus surgery vs. surgery alone for LAEC, thereby providing more precise and reliable evidence for clinical practice.

## Materials and methods

This meta-analysis was prospectively registered on the PROSPERO International Prospective Register of Systematic Reviews (Registration number: CRD420251251423). The conduct of this systematic review adhered to the Preferred Reporting Items for Systematic Reviews and Meta-Analyses Protocols (PRISMA-P) 2015 statement. Additionally, we followed the 12-item PRISMA extension guideline.

### Eligibility criteria

#### Inclusion criteria

(1) Study type: Published RCTs from any country. (2) Participants: Adult patients (age ≥18 years) with pathologically confirmed LAEC (clinical stage cT1-4a, N0-1, M0). The N0-1 criteria reflect the inclusion protocols of the original landmark trials (e.g., CROSS and NEOCRTEC5010), in which N2-3 disease was often excluded due to a higher risk of occult distant metastases and inconsistent neoadjuvant approaches at the time of trial design. (3) Intervention: The experimental group received standard nCRT followed by radical esophagectomy. (4) Comparator: The control group underwent radical esophagectomy alone (surgery alone). (5) Outcomes: Studies must report at least one of the following core outcomes: overall survival (OS), disease-free survival (DFS), or R0 resection rate, with data available for extraction and pooled analysis.

#### Exclusion criteria

(1) Non-randomized studies (e.g., observational studies, case reports, reviews, conference abstracts). (2) Studies including patients with distant metastasis (M1). (3) Intervention mismatch (e.g., neoadjuvant chemotherapy or radiotherapy alone in the experimental group). (4) Studies with unavailable data, duplicate publications, or insufficient information.

#### Outcome definitions

Overall Survival (OS): The time from randomization to death from any cause. We extracted the hazard ratio (HR) and its 95% confidence interval (CI) for pooled analysis.

Disease-Free Survival (DFS): The time from randomization to disease recurrence, progression, or death from any cause. We extracted the HR and its 95% CI for pooled analysis.

R0 Resection Rate: Complete microscopic resection with no tumor cells at any margin confirmed by postoperative pathological examination. We extracted the number of patients achieving R0 resection and the total number of patients undergoing surgery in each group to calculate the odds ratio (OR) for pooling.

### Search strategy

#### Data sources

We systematically searched the PubMed, Embase, Cochrane Central Register of Controlled Trials (CENTRAL), and Web of Science Core Collection databases.

#### Search terms

English search terms combined Medical Subject Headings (MeSH) and free-text words, including: “Esophageal Neoplasms”[Mesh], “Esophageal Cancer”, “Neoadjuvant Therapy”[Mesh], “Chemoradiotherapy, Adjuvant”[Mesh], “Preoperative Chemoradiation”, “Esophagectomy”[Mesh], “Surgery”, “Randomized Controlled Trial”[Mesh]. Boolean operators (AND, OR) were used to construct the search strategy.

#### Timeframe

The search covered all relevant literature published from the inception of each database until March 31, 2025, with no language restrictions.

#### Study selection and data extraction

Two investigators independently performed study selection and data extraction, followed by cross-verification. Disagreements were resolved through discussion or arbitration by a third investigator. Extracted data primarily included: first author, publication year, study design, sample size, patient baseline characteristics (age, clinical stage, histology), intervention details (chemotherapy regimen, radiotherapy dose, surgical approach), follow-up duration, and data for OS (HR and 95% CI), DFS (HR and 95% CI), and raw event numbers for R0 resection.

#### Quality assessment

The methodological quality of the included RCTs was assessed using the Cochrane Risk of Bias tool 2.0 (ROB 2.0). This tool evaluates five domains: (1) randomization process; (2) deviations from intended interventions; (3) missing outcome data; (4) measurement of the outcome; and (5) selection of the reported result. The risk of bias in each domain was judged as “low risk,” “some concerns,” or “high risk,” and an overall risk of bias judgment for each study was determined accordingly.

#### Statistical methods

Meta-analysis was performed using Stata software version 18.0. For time-to-event data (OS, DFS), the extracted HRs and their 95% CIs were pooled. For dichotomous data (R0 resection rate), ORs and their 95% CIs were pooled. The I^2^ statistic was used to assess heterogeneity among studies. I^2^ ≤ 25% was defined as low heterogeneity, 25% < I^2^ ≤ 50% as moderate heterogeneity, and I^2^ > 50% as high heterogeneity. A fixed-effect model was used for pooling when low or moderate heterogeneity was present; a random-effects model was used when high heterogeneity was present, and subgroup analyses were conducted to explore sources of heterogeneity. Sensitivity analysis was performed by sequentially omitting individual studies to assess the robustness of the results. If 10 or more studies were included for an outcome, publication bias was assessed using funnel plots and Egger's test. All statistical tests were two-sided, and a *P* value < 0.05 was considered statistically significant.

## Results

### Literature search results

**Figure 1:**
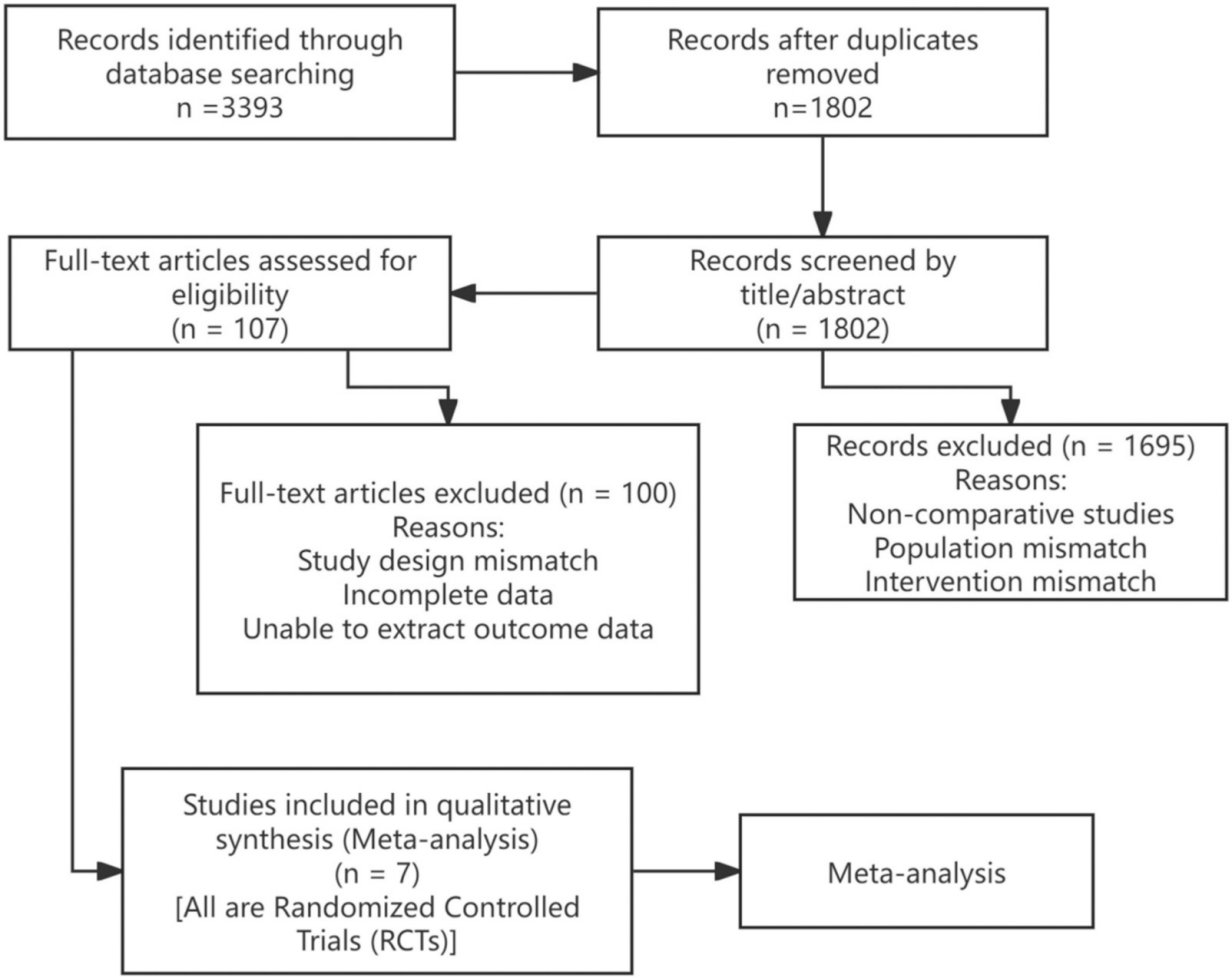
PRISMA flow diagram.

The initial systematic search of databases (PubMed, Embase, Cochrane Library, etc.), supplemented by manual searches of relevant reviews and reference lists, yielded 3,393 potentially relevant records. After removing duplicates using EndNote software, 1,802 records remained for initial screening. Based on title and abstract review, 1,695 records were excluded for clear ineligibility (e.g., non-comparative studies, wrong patient population, wrong intervention), leaving 107 records for full-text retrieval and detailed assessment. After careful full-text review, 100 records were further excluded due to mismatched study design, incomplete data, or inability to extract required outcome data. Finally, seven eligible RCTs were included in this meta-analysis. The detailed study selection process is shown in Figure 1 (PRISMA flow diagram).

### Characteristics of included studies

A total of seven randomized controlled trials (RCTs), encompassing 1,168 participants, were included. The baseline characteristics of the included studies are presented in Table 1. Among the seven included RCTs, five studies reported OS and DFS with extractable hazard ratios, and four studies reported R0 resection rates. The specific studies contributing to each outcome are indicated in Table 1.

**Table 1:** Baseline characteristics of included studies.

Study (year)	Study design	Group	Intervention	Regimen details	Age (years)	Follow-up (months)	Outcome measures
Walsh TN 1996 (10)	RCT	Neoadjuvant CRT + Surgery	Fluorouracil+Cisplatin chemotherapy+Radiotherapy+Surgery	Fluorouracil 15 mg/kg/day, IV infusion over 16 h for 5 days; Cisplatin 75 mg/m^2^, IV infusion over 8 h (Day 7, pre-hydration with 2L 0.9% saline the day before chemotherapy); 2 cycles of chemotherapy (Weeks 1 & 6); Radiotherapy 40 Gy/15 fractions (2.67 Gy/fraction) concurrent with Cycle 1 chemotherapy	Median 65	Median 10	Overall survival, 3-year survival rate, Pathological complete response rate, Lymph node positivity/metastasis rate, Treatment-related toxicity (WHO grading), Postoperative complications (Respiratory/Cardiac/Anastomotic leak, etc.), In-hospital mortality
		Surgery alone	Surgery alone	-	Median 65	Median 8	
Bosset JF 1997 (11)	RCT	Neoadjuvant CRT + Surgery	Cisplatin+Radiotherapy+Surgery	Cisplatin 80 mg/m^2^, IV infusion on days 0–2 prior to radiotherapy; Radiotherapy 37 Gy (in 2 courses: 18.5 Gy/5 fractions, 3.7 Gy/fraction per course, with a 2-week interval between courses); Surgery 2–4 weeks post-radiotherapy	Median 56.7	Median 55.2	Overall survival, Disease-free survival, Local recurrence-free survival, Time to distant metastasis, Curative resection rate, Postoperative mortality, Treatment-related toxicity (Nausea/Neutropenia, etc.)
		Surgery alone	Surgery alone	-	Median 56.6	Median 55.2	
van Hagen P 2012 (12)	RCT	Neoadjuvant CRT + Surgery	Carboplatin+Paclitaxel chemotherapy+Radiotherapy+Surgery	Carboplatin AUC 2 mg/mL·min, Paclitaxel 50 mg/m^2^, IV infusion, once weekly for 5 weeks; Radiotherapy 41.4 Gy/23 fractions (1.8 Gy/fraction) concurrent with chemotherapy	Median 60	Median 45.4 (in survivors)	Overall survival, Disease-free survival, R0 resection rate, Pathological complete response rate, Treatment-related adverse events (Hematological/Non-hematological), Postoperative complications (Pulmonary/Cardiac/Anastomotic leak, etc.), In-hospital mortality
		Surgery alone	Surgery alone	-	Median 60	Median 45.4 (in survivors)	
Tepper J 2008 (13)	RCT	Neoadjuvant CRT + Surgery	Cisplatin+Fluorouracil chemotherapy+Radiotherapy+Surgery	Cisplatin 100 mg/m^2^, IV infusion over 30 min (Days 1 & 29); Fluorouracil 1,000 mg/m^2^/day, continuous IV infusion over 96 h (Days 1–4 & 29–32); Radiotherapy 50.4 Gy/28 fractions (1.8 Gy/fraction) concurrent with chemotherapy	Mean 60.9	Median 72 (6 years)	Overall survival, Progression-free survival, Pathological response rate (Complete/Partial), Treatment-related toxicity (Grade 3–5), Postoperative complications (Infection/Respiratory failure, etc.), Site of recurrence (Local/Distant)
		Surgery alone	Surgery alone	-	Mean 61.9	Median 70.8 (5.9 years)	
Shapiro J 2015 (14)	RCT	Neoadjuvant CRT + Surgery	Carboplatin+Paclitaxel chemotherapy+Radiotherapy+Surgery	Identical to van Hagen P 2012 (6) (Long-term results of the same CROSS trial)	Median 60	Median 84.1 (in survivors)	Overall survival, Progression-free survival, Locoregional progression rate, Distant progression rate, R0 resection rate, Pathological complete response rate, Treatment-related toxicity
		Surgery alone	Surgery alone	-	Median 60	Median 84.1 (in survivors)	
Yang H 2018 (15)	RCT	Neoadjuvant CRT + Surgery	Vinorelbine+Cisplatin chemotherapy+Radiotherapy+Surgery	Vinorelbine 25 mg/m^2^, IV bolus (Days 1 & 8); Cisplatin 75 mg/m^2^ IV infusion over 3 h (Day 1) OR 25 mg/m^2^ IV infusion over 2 h (Days 1–4); 2 cycles of chemotherapy (one cycle every 3 weeks); Radiotherapy 40 Gy/20 fractions (2.0 Gy/fraction) concurrent with Cycle 1 chemotherapy	Median 60	Median 41.0 (in survivors)	Overall survival, Disease-free survival, R0 resection rate, Pathological complete response rate, Treatment-related adverse events (CTCAE v3.0 grading), Postoperative complications (Arrhythmia/Pulmonary infection, etc.), Peri-treatment mortality
		Surgery alone	Surgery alone	-	Median 59	Median 34.6 (in survivors)	
Liu S 2020 (16)	RCT	Neoadjuvant CRT + Surgery	Vinorelbine+Cisplatin chemotherapy+Radiotherapy+Surgery	Identical to Yang H 2018 (2) (Recurrence analysis of the same NEOCRTEC5010 trial)	Median 57	Median 51.9 (in survivors)	Recurrence rate, Locoregional recurrence-free survival, Distant metastasis-free survival, Time to recurrence, Site of recurrence (Anastomotic/Regional lymph nodes/Distant organs, etc.), Prognostic factors (Resection margin/Pathological N stage, etc.)
		Surgery alone	Surgery alone	-	Median 57	Median 51.9 (in survivors)	

OS/DFS data available from Walsh 1996 (10), Bosset 1997 (11), van Hagen 2012 (12)/Shapiro 2015 (14), Tepper 2008 (13), Yang 2018 (15). R0 data available from Bosset 1997 (11), van Hagen 2012 (12)/Shapiro 2015 (14), Yang 2018 (15), Liu 2020 (16). CTCAE, common terminology criteria for adverse events (National Cancer Institute, version 3.0).

### Quality assessment

**Figure 2:**
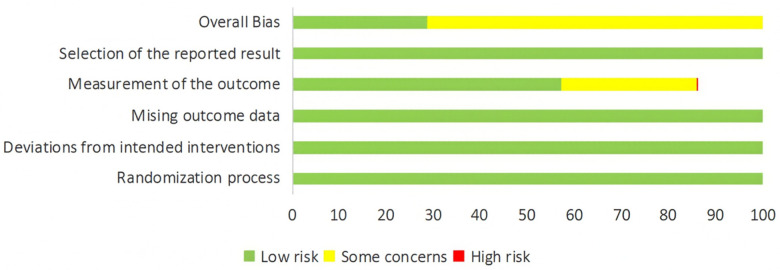
Literature quality assessment.

The seven included studies were assessed using the Risk of Bias (ROB) 2.0 tool across six domains: randomization process, deviations from intended interventions, missing outcome data, measurement of the outcome, selection of the reported result, and overall bias. The results indicated a low risk of bias across all domains. This finding demonstrates that the studies featured standardized design, rigorous execution, and strong data reliability, indicating an overall high quality. The quality assessment is illustrated in Figure 2.

### Meta-analysis results

#### Overall survival (OS)

**Figure 3:**
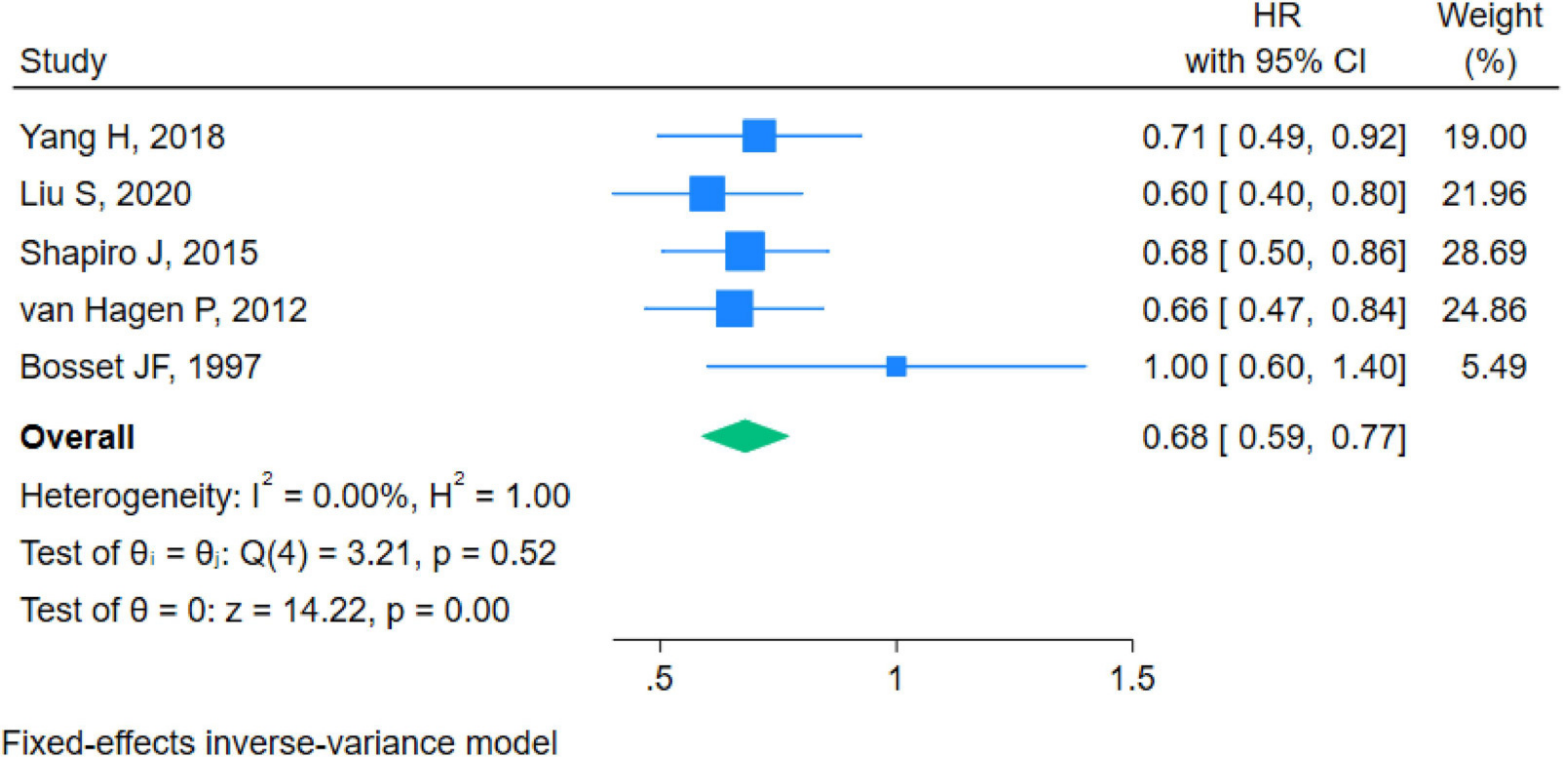
Forest plot for overall survival.

The forest plot analysis for OS demonstrated a pooled hazard ratio (HR) of 0.68 (95% CI 0.59–0.77). Heterogeneity testing indicated I^2^ = 0.00%, H^2^ = 1.00, with Q(4) = 3.21 and *p* = 0.52, suggesting no significant heterogeneity. A fixed-effect inverse variance model was applied. The test results showed z = 14.22 and *p* < 0.001, indicating that neoadjuvant chemoradiotherapy combined with surgery significantly improved overall survival compared to surgery alone (Figure 3).

#### DFS

**Figure 4:**
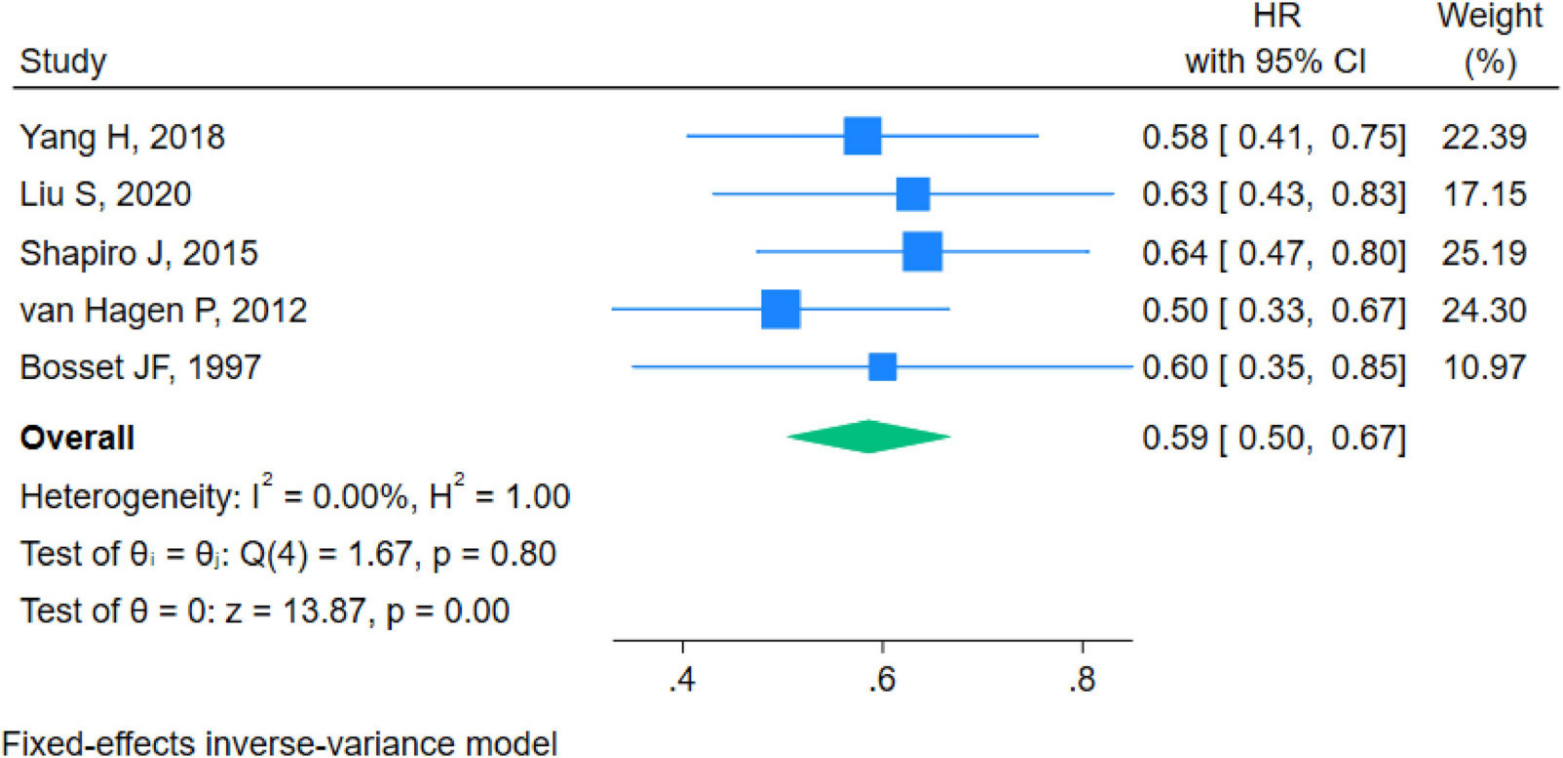
Forest plot for DFS.

The forest plot analysis for disease-free survival demonstrated a pooled hazard ratio (HR) of 0.59 (95% CI 0.50–0.67). Heterogeneity testing yielded I^2^ = 0.00%, H^2^ = 1.00, Q(4) = 1.67, and *p* = 0.80, indicating no significant heterogeneity. A fixed-effect inverse variance model was therefore applied. The test results showed z = 13.87 and *p* < 0.001, indicating that neoadjuvant chemoradiotherapy combined with surgery significantly improved disease-free survival compared with the direct surgery group (Figure 4).

#### RO

**Figure 5:**
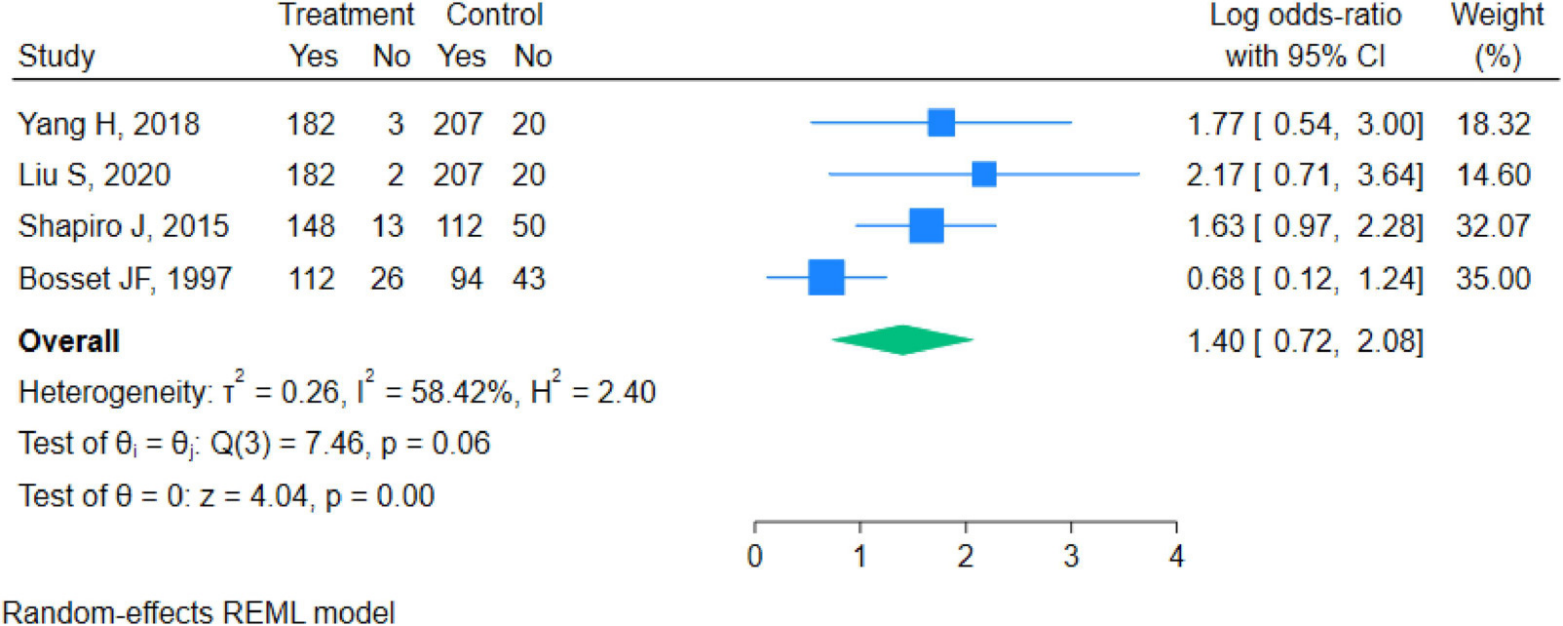
Forest plot for R0 resection.

Analysis of the R0 resection rate forest plot revealed a pooled odds ratio (OR) of 1.40 (95% CI: 0.72–2.08). Heterogeneity testing showed T^2^ = 0.26, I^2^ = 58.42%, H^2^ = 2.40, and Q(3) = 7.46 with *p* = 0.06, indicating moderate heterogeneity. A random-effects REML model was applied. The test results demonstrated z = 1.00 and *p* = 0.32, indicating that the difference in R0 resection rates between the two groups was not statistically significant (Figure 5).

#### Publication bias

**Figure 6:**
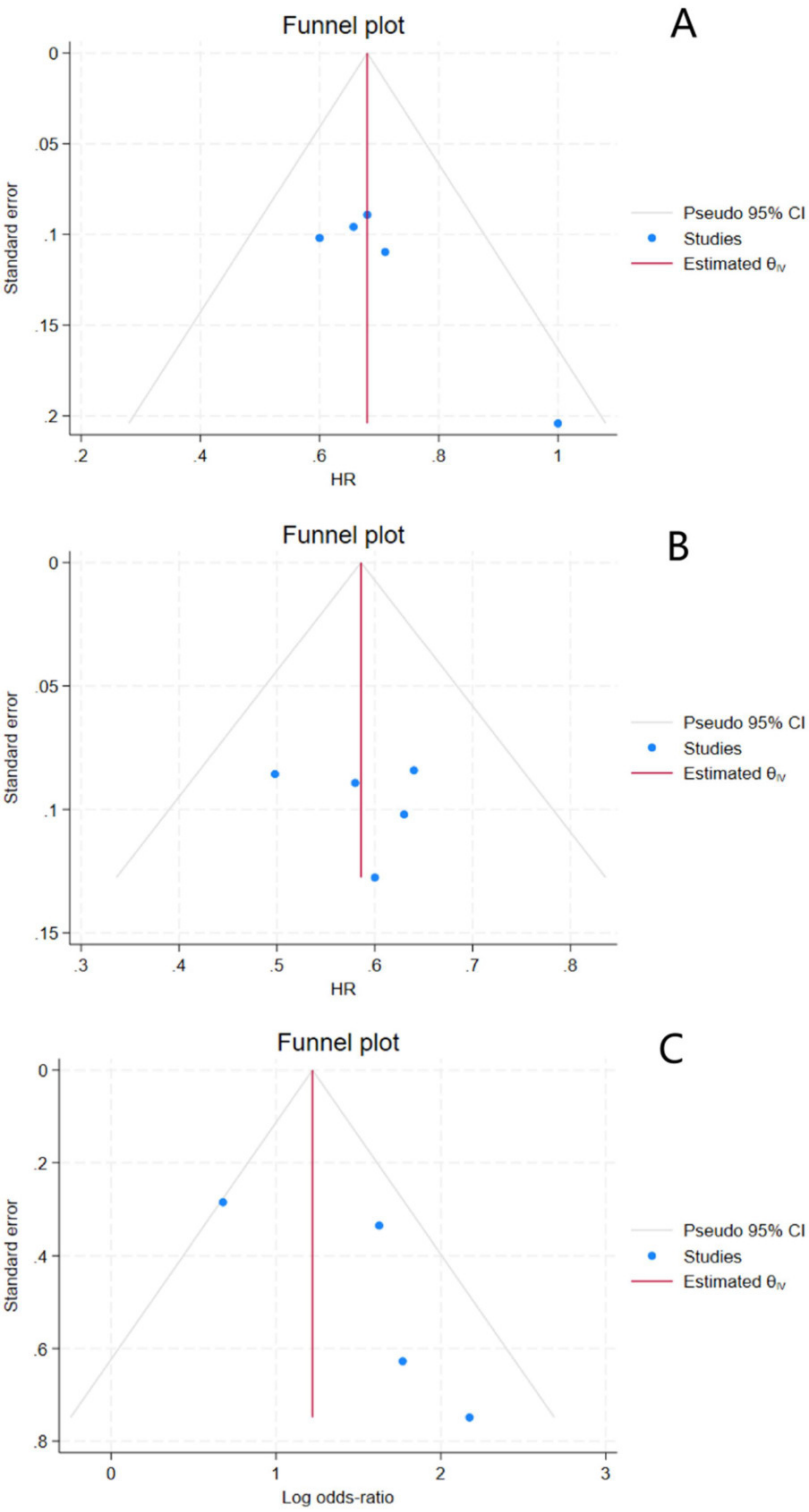
Funnel plot for publication bias. **(A)** Overall survival; **(B)** Disease-free survival; **(C)** R0 resection rate.

Funnel plots were constructed to assess potential publication bias (Figure 6).

In the funnel plot for overall survival (OS), the individual study estimates were distributed roughly symmetrically around the pooled effect size (HR = 0.68) and within the pseudo 95% confidence limits. The plot exhibited an approximately symmetrical funnel shape, and no obvious asymmetry was observed visually. This suggests a low likelihood of significant publication bias for OS.

The funnel plot for disease-free survival (DFS) showed a relatively uniform distribution of study points on both sides of the effect size axis (HR). The funnel plot contour was largely symmetrical, with no apparent clustering on one side. These findings indicate a minimal risk of substantial publication bias for this outcome.

Conversely, the funnel plot for the R0 resection rate demonstrated some asymmetry. Several study points were scattered outside the pseudo 95% confidence limits and skewed to one side of the effect size axis (Log odds-ratio). The overall shape of the funnel was not entirely symmetrical, suggesting the possible presence of publication bias.

In summary, the funnel plots for OS and DFS showed good symmetry, whereas the funnel plot for the R0 resection rate indicated potential publication bias. This asymmetry may be associated with the non-publication or non-retrieval of studies with negative results (i.e., those showing no significant improvement in R0 resection rate with the combined therapy). This potential bias should be considered when interpreting the corresponding findings.

#### Sensitivity analysis

Sensitivity analyses for OS and DFS demonstrated that the heterogeneity (I^2^) remained at a low level (<10%) after the sequential omission of any single study. The direction of the pooled hazard ratios (HRs) and their statistical significance (*P* < 0.001) remained unchanged, indicating robust results. For R0 resection, sensitivity analysis by sequentially omitting individual studies showed that heterogeneity fluctuated depending on the included studies. When the study by Bosset JF et al. (1997) was excluded, heterogeneity decreased to I^2^ = 19%. In all iterations, the pooled OR remained consistent in direction (favoring nCRT) but, as noted above, the difference was not statistically significant (*P* = 0.32). This confirms the robustness of the finding that nCRT does not significantly improve the R0 resection rate.

#### Subgroup analysis

To further explore the sources of heterogeneity in the R0 resection rate and to assess treatment effects across populations with different characteristics, subgroup analyses were performed based on key study features. These analyses used the pooled odds ratio (OR) and its 95% confidence interval (CI) as the effect measure, stratified by “tumor subtype” and “region/study population”.

**Figure 7:**
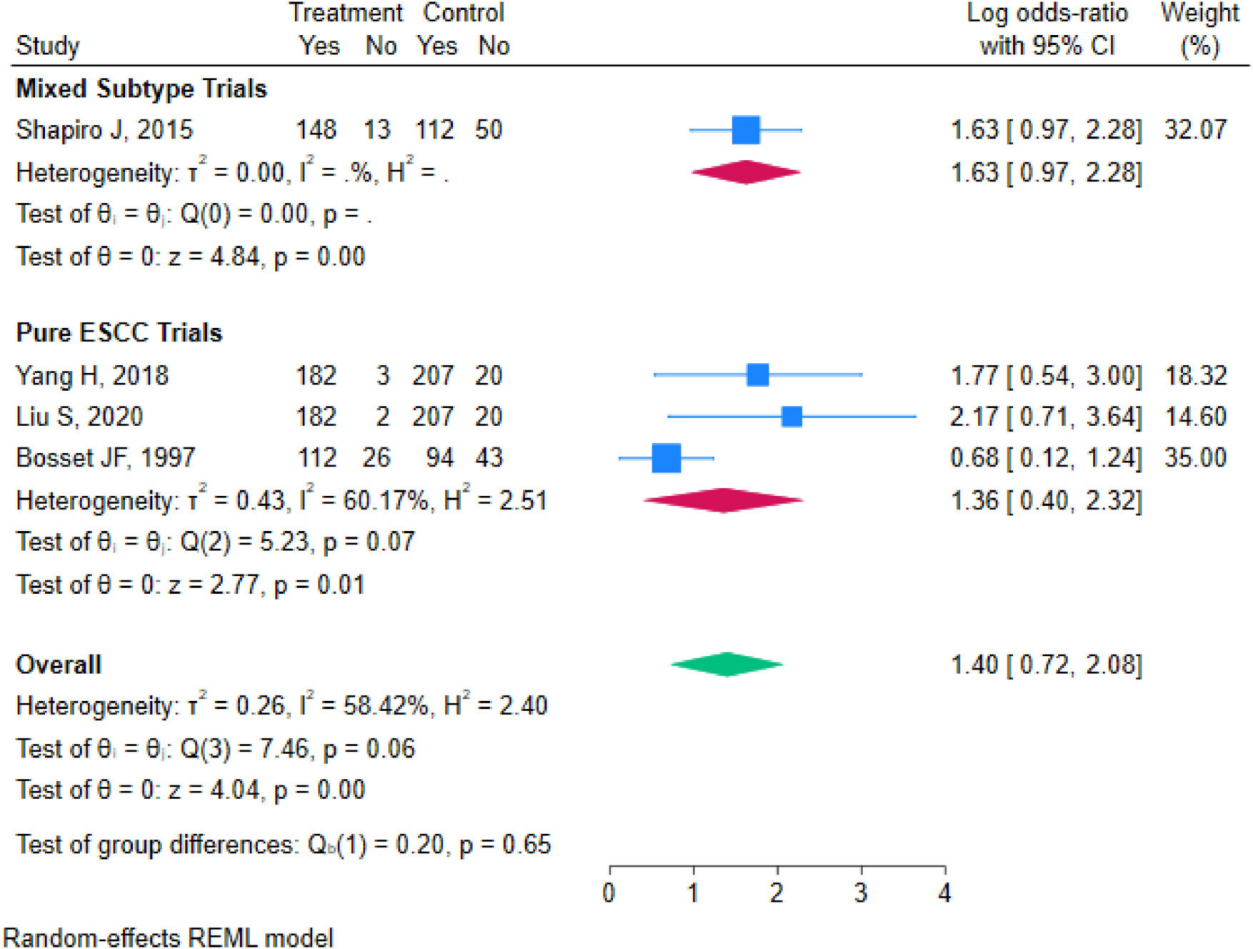
Forest plot of R0 resection rate stratified by tumor subtype.

Stratified by Tumor Subtype: Studies were categorized into a “Pure Esophageal Squamous Cell Carcinoma (ESCC) Subgroup” and a “Mixed Histology Subgroup” based on pathology. The pure ESCC subgroup included three studies (Yang H 2018, Liu S 2020, Bosset JF 1997) with 1,044 patients. This subgroup exhibited moderate heterogeneity (I^2^ = 60.17%, *τ*^2^ = 0.43, Q = 5.23, *p* = 0.07), and a random-effects model was applied. The pooled analysis showed a significantly higher R0 resection rate in the neoadjuvant chemoradiotherapy plus surgery group compared to the surgery-alone group (OR=1.36, 95% CI 0.40–2.32, *p* = 0.01). The mixed histology subgroup (including adenocarcinomas and adenosquamous carcinomas) was represented by the Shapiro 2015 (14) study (long-term follow-up of the CROSS trial), which included 366 patients; the van Hagen 2012 (12) report was not separately analyzed to avoid duplicate counting of the same cohort. The pooled OR was 1.63 (95% CI 0.97–2.28, *p* < 0.001). The test for subgroup differences indicated no statistically significant heterogeneity between subgroups (Qb = 0.20, *p* = 0.65), suggesting that tumor subtype was not a major source of heterogeneity for this outcome (Figure 7).

**Figure 8:**
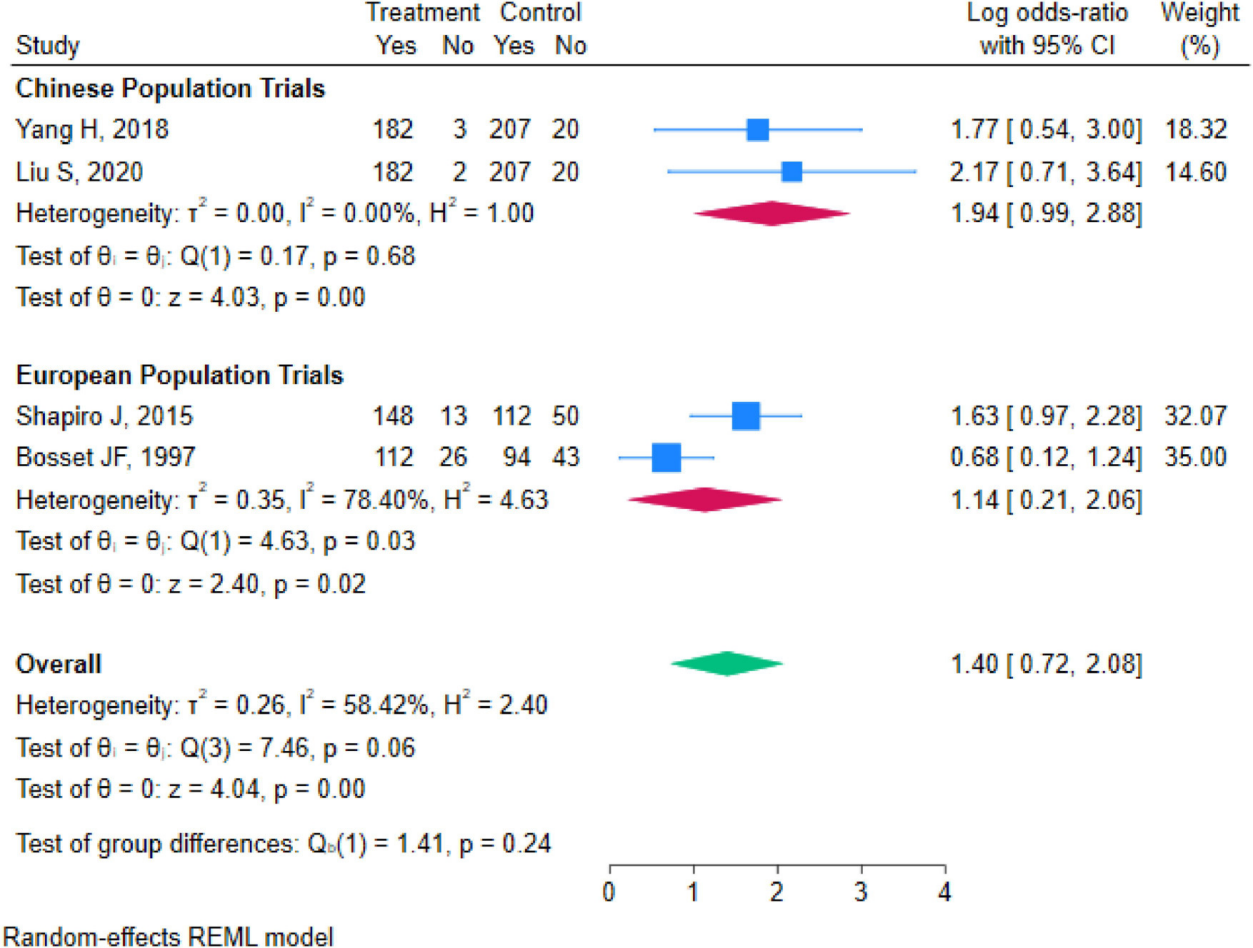
Forest plot of R0 resection rate stratified by region/study population.

Stratified by Region/Study Population: Studies were divided into a “Chinese Population Subgroup” and a “European Population Subgroup” based on the region of conduct and population characteristics. The Chinese population subgroup included two studies (Yang H 2018, Liu S 2020) with 860 patients. It showed no heterogeneity (I^2^ = 0.00%, Q = 0.17, *p* = 0.68), and a fixed-effects model was used. The pooled analysis demonstrated that combined therapy significantly increased the R0 resection rate (OR=1.94, 95% CI 0.99–2.88, *p* < 0.001). The European population subgroup included two studies (Shapiro J 2015, Bosset JF 1997) with 654 patients and exhibited high heterogeneity (I^2^ = 78.40%, *τ*^2^ = 0.35, Q = 4.63, *p* = 0.03), necessitating a random-effects model. The pooled OR was 1.14 (95% CI 0.21–2.06, *p* = 0.02). The test for subgroup differences was not statistically significant (Qb = 1.41, *p* = 0.24), indicating that region/population was not a significant modifier of heterogeneity. However, the effect size in the Chinese population subgroup (OR=1.94) was numerically higher than that in the European subgroup (OR=1.14). This difference might be associated with factors such as the extent of lymph node dissection, pathological assessment standards, or ethnic variations, warranting attention in future research (Figure 8).

## Discussion

This study systematically evaluated the efficacy of nCRT plus surgery vs. surgery alone based on 7 RCTs involving 1,168 patients with LAEC. The results demonstrate that nCRT plus surgery significantly improves OS (HR = 0.68) and DFS (HR = 0.59) compared to surgery alone. It also shows a trend toward a higher R0 resection rate, although this difference did not reach statistical significance (OR=1.40, 95% CI 0.72–2.08, *P* = 0.32). Assessment for publication bias suggested potential bias for the R0 resection rate. Sensitivity analyses confirmed that the results for OS and DFS were highly robust. Although the result for the R0 resection rate was influenced by specific studies, leading to fluctuations in heterogeneity, the direction of the effect remained stable. Subgroup analyses further revealed that neither tumor pathological subtype (pure squamous cell carcinoma vs. mixed type) nor geographic population (China vs. Europe) were major statistical sources of heterogeneity for the R0 resection rate. However, the treatment effect trend was more pronounced in the Chinese population.

Regarding the therapeutic mechanism, the advantage of nCRT lies in its multimodal synergistic effect (17). Radiotherapy directly kills local tumor cells, reduces tumor volume, and downstages the disease. Concurrent chemotherapy not only acts as a radiosensitizer but also treats potential systemic micrometastases at an early, preoperative stage (18, 19). This dual effect of “downstaging” and “controlling micrometastases” creates more favorable conditions for subsequent radical surgery. This is reflected in a numerically higher R0 resection rate, although the difference was not statistically significant in our pooled analysis. Successful R0 resection is a cornerstone for the long-term prognosis of esophageal cancer patients (20). This study confirms that achieving a higher R0 resection rate through nCRT translates into significant OS and DFS benefits. This forms a complete logical chain from effective neoadjuvant therapy to successful surgical radicalization, ultimately improving survival outcomes.

The findings of this study are consistent with recent high-quality meta-analyses, which report that nCRT provides definitive survival benefits for resectable esophageal cancer (21, 22). However, the depth of this study lies not only in confirming the survival advantage but also in focusing on the R0 resection rate as a key surgical endpoint. Through in-depth heterogeneity exploration (subgroup analysis), we found that although the effect sizes differed numerically between populations (China and Europe), the benefit of combined therapy in increasing the R0 resection rate was generally consistent across the subgroups we examined, although the precision of the effect estimates varied, as reflected by the confidence intervals. This provides more concrete evidence supporting the broad application of nCRT in different healthcare settings. Furthermore, the literature included in this study is updated, incorporating long-term follow-up data from recent trials such as NEOCRTEC5010, making the evidence more contemporary.

From a clinical perspective, patients with esophageal squamous cell carcinoma (particularly in Asian populations where ESCC predominates) and those with good performance status may derive the greatest absolute benefit from nCRT, as suggested by the numerically higher R0 resection effect in the Chinese subgroup (OR=1.94) and the pure ESCC subgroup (OR=1.36), although these differences were not statistically significant.

This study has several strengths. First, the search strategy was systematic and comprehensive, covering multiple major databases, which minimized the risk of omitting relevant studies. Second, all included studies were RCTs with relatively high methodological quality. They were rigorously evaluated using the ROB 2.0 tool, ensuring the reliability of the primary evidence. Third, the statistical analysis was rigorous. In addition to conventional pooled analysis, thorough sensitivity and subgroup analyses were performed for the heterogeneous outcome (R0 resection rate), enhancing the robustness and interpretative depth of the conclusions.

Simultaneously, this study has certain limitations. First, heterogeneity was present for some outcomes. While OS and DFS showed no heterogeneity, the R0 resection rate exhibited moderate heterogeneity (I^2^ = 58.42%). Although subgroup analyses did not identify a statistically significant influence of tumor subtype or geography, they did not fully explain the source of heterogeneity. Unmeasured factors likely contributed, including variations in chemotherapy regimens (e.g., cisplatin/fluorouracil vs. carboplatin/paclitaxel vs. vinorelbine/cisplatin), radiotherapy doses (range 37–50.4 Gy), fractionation schedules, surgical techniques (e.g., extent of lymphadenectomy, transthoracic vs. transhiatal approach), and pathological assessment criteria for R0 status. These factors were not uniformly reported across trials, limiting our ability to explore them quantitatively. Furthermore, the subgroup analyses for R0 resection included only two to three studies per subgroup; therefore, the non-significant subgroup difference tests (*p* = 0.65 for tumor subtype, *p* = 0.24 for region) should be interpreted with caution, as the limited statistical power may have failed to detect true differences Second, there is a possibility of publication bias. The funnel plot asymmetry for the R0 resection rate suggests the potential for unpublished negative results, which might lead to a slightly optimistic estimate of this effect. Third, follow-up durations varied across the included studies, ranging from months to years, which might introduce some inconsistency in the assessment of very long-term survival outcomes.

Additionally, meta-regression for the primary outcome (OS) was not feasible due to the limited number of studies (<10), as recommended by Cochrane guidelines, which may have precluded the identification of specific sources of between-study heterogeneity.

Future research could further conduct individualized meta-analyses or network meta-analyses focusing on different pathological types (e.g., squamous cell carcinoma vs. adenocarcinoma) and different nCRT regimens (e.g., chemotherapeutic drug combinations, radiation doses) to identify the optimal treatment strategy. Concurrently, more studies are needed to focus on the long-term quality of life impact and specific adverse effects of nCRT.

This meta-analysis indicates that for patients with LAEC, the comprehensive treatment strategy of nCRT plus surgery significantly improves the R0 resection rate compared to surgery alone. This translates into significant improvements in overall survival and disease-free survival, without increasing treatment-related mortality. Therefore, nCRT plus surgery should be considered one of the standard treatment options for locally advanced esophageal cancer. In clinical practice, nCRT is recommended for eligible patients to achieve better radical effects and long-term survival.

## Data Availability

The raw data supporting the conclusions of this article will be made available by the authors, without undue reservation.
